# Large and linked in scientific publishing

**DOI:** 10.1186/2047-217X-1-1

**Published:** 2012-07-12

**Authors:** Laurie Goodman, Scott C Edmunds, Alexandra T Basford

**Affiliations:** 1 GigaScience, BGI-Hong Kong Co. Ltd, 16 Dai Fu Street, Tai Po Industrial Estate, NT, Hong Kong

## Abstract

We are delighted to announce the launch of *GigaScience*, an online open-access journal that focuses on research using or producing large datasets in all areas of biological and biomedical sciences. *GigaScience* is a new type of journal that provides standard scientific publishing linked directly to a database that hosts all the relevant data. The primary goals for the journal, detailed in this editorial, are to promote more rapid data release, broader use and reuse of data, improved reproducibility of results, and direct, easy access between analyses and their data. Direct and permanent connections of scientific analyses and their data (achieved by assigning all hosted data a citable DOI) will enable better analysis and deeper interpretation of the data in the future.

## *GigaScience* goals and scope

“Big-data” science has been growing by leaps and bounds over the last decade. While data availability has provided myriad new opportunities for research, full use of these data across all the life sciences requires more focused mechanisms to reach the promise of community resource projects. This is especially true for smaller labs that do not have the computational facilities to take full advantage of such resources, which are intended to speed work and provoke novel hypotheses for testing.

Unique to *GigaScience*—and essential to achieving community-wide goals for taking full advantage of large, sharable datasets across the board—is the creation of a system that more easily links publications to their complete datasets, provides citable, countable credit for data producers, and makes data more accessible and useable to the entire life-science community. To address some of these issues, we have devised a new journal model that integrates manuscript publication with a database that houses and provides tools for the data used in these publications. The database, *Giga*DB, provides all included datasets with reference-section citable DOIs; *Giga*DB data have already been referenced in several top tier journals (for details, see [1]).

Additionally, although the “omics” communities have well-established data sharing mechanisms and standards, there are many fields that produce equal if not larger data sets that are not readily sharable and that require more work for establishing standards and sharing. Thus, *GigaScience* and *Giga*DB are especially interested in supporting non-omics type research, as these typically have sharable data but no broadly accepted public repositories or completely established means to promote the widest free sharing of data.

We do want to stress, as this has been an issue raised by many, that if there are permanent or community-agreed upon databases available (e.g. NCBI, EBI databases, and similar), we *require* that the data be submitted to those as well. The reasoning is simple: broader data sharing and permanence means broader data usage—and usage is key.

## Peer review

In addition to trying to make the availability and use of data associated with our papers more transparent, we are also focusing on doing the same with our peer-review model. We are using an opt-out open peer review system, a system that is becoming increasingly accepted in the medical community. Reviewers’ names are included with their reviews unless a reviewer has reasons not to be named and opts out. The reviews will be available in the pre-publication history section of our papers so that the entire set of comments and history can be seen by anyone interested in the additional insight that may come from the behind-the-scenes discussions surrounding the review.

We also are taking steps to avoid what might be called the “science *du jour*’ phenomenon, where reviewers might indicate the work is sound but not of ‘interest’. At *GigaScience*, the Editors, in consultation with our Editorial Board when needed, will make the overall decision on whether the work is of interest. Editorial decisions in this regard will be based on scope and relative amount of data created or used (see http://blogs.openaccesscentral.com/blogs/gigablog/?page=2 for information on what constitutes “big data”). Assessing the potential impact of research is extremely difficult and can be subjective, and there are huge technical challenges in assessing data supporting large-scale research studies. What is much easier to do is to assess transparency and compliance with best-practice guidelines for reporting and presenting data. Our reviewers are specifically asked to report on these issues, and all data are given what we refer to as a ‘sanity check’ by our curators to determine if the data themselves are sound. Thus, peer review at *GigaScience* focuses on whether the biological conclusions are well supported, and if the data are sound and follow appropriate community standards. The level of ‘interest’ of the work will ultimately be determined via the best means of determining data and research quality: its use by the community.

## In this issue

Our launch issue contains a variety of papers that highlight several of the aims for publications in *GigaScience*. This issue shows two types of the journal’s research articles: standard Research articles and Technical Notes. Standard research papers present novel data and analyses, exemplified here by an article from the laboratory of one of the members of our excellent editorial board and that describes a novel analysis pathway and creates a unique methylomic resource [2]. The work by Daniel McDonald *et al.*[3] in this issue is a technical note and presents a novel data format that facilitates the interoperability of bioinformatics tools.

The issue also includes several Commentaries, including one that is associated with a research paper in this issue: Jonathan Eisen’s commentary on ‘badomics’ terminology [4], which focuses on the explosion of “omes” (good and bad) noted in the McDonald *et al.* study. The first article in our thematic series covering the best practices in genomics research, done in concert with the Genomic Standards Consortium, is a commentary detailing several of the challenges for developing community standards and data-sharing policies [5], which are key to maximizing data reuse. Furthermore, the issues surrounding the handling of large-scale data are not just affecting the omics community, and a more broadly focused commentary on data sharing for neuroimaging [6] highlights this as well as the fact that our scope also covers areas such as neuroscience, imaging, biomedicine and ecology. Along a different vein, we have a commentary promoting the development of a digital immune system to serve as a global sequencing based pathogen monitoring system as increasing sensitivity and decreasing costs of sequencing technologies increase utility of sequencing as a sensor [7].

This issue also has several Reviews, which are typically more in-depth than commentaries and which serve to provoke forward-thinking with regards to what steps are required next to advance projects or overcome large-data handling issues. One of the reviews focuses on the difficulties of and potential solutions for sustainable archiving of the ever-growing amount of sequencing data [8]. Another review is a white paper from the G10K vertebrate project that details the strategies and best practices for sample collection [9]. The last raises the idea of developing ‘Genome Observatories’ [10] to provide a digital means to characterize whole ecosystems with the purpose of promoting more contextual information to accompany genomic data.

We hope you enjoy this issue. We encourage you to contact any of the editors to begin conversations about specific needs in your research communities for promoting large-data access, sharing, use, and reuse.

## Competing interests

All authors are employees of *GigaScience* and BGI.

## Authors’ contributions

All authors have been working on *GigaScience* and *Giga*DB, and have contributed to this editorial. All authors read and approved the final manuscript.
